# Understanding the COVID-19 Pandemic in Nursing Homes (Aragón, Spain): Sociodemographic and Clinical Factors Associated With Hospitalization and Mortality

**DOI:** 10.3389/fpubh.2022.928174

**Published:** 2022-07-07

**Authors:** Isabel Aguilar-Palacio, Lina Maldonado, Iván Marcos-Campos, Sara Castel-Feced, Sara Malo, Carlos Aibar, MªJosé Rabanaque

**Affiliations:** ^1^Preventive Medicine and Public Health Department, University of Zaragoza, Zaragoza, Spain; ^2^Instituto de Investigación Sanitaria de Aragón, Instituto de Investigación Sanitaria de Aragón (IIS), Zaragoza, Spain; ^3^Grupo de Investigación en Servicios Sanitarios de Aragón (GRISSA), Instituto de Investigación Sanitaria de Aragón (IIS), Zaragoza, Spain; ^4^Department of Applied Economics, Economic History and Public Economics, University of Zaragoza, Zaragoza, Spain

**Keywords:** COVID-19, inequalities, hospitalization, mortality, nursing home

## Abstract

Old people residing in nursing homes have been a vulnerable group to the coronavirus disease 2019 (COVID-19) pandemic, with high rates of infection and death. Our objective was to describe the profile of institutionalized patients with a confirmed COVID-19 infection and the socioeconomic and morbidity factors associated with hospitalization and death. We conducted a retrospective cohort study including data from subjects aged 65 years or older residing in a nursing home with a confirmed COVID-19 infection from March 2020 to March 2021 (4,632 individuals) in Aragón (Spain). We analyzed their sociodemographic and clinical profiles and factors related to hospitalization and mortality at 7, 30, and 90 days of COVID-19 diagnosis using logistic regression analyses. We found that the risk of hospitalization and mortality varied according to sociodemographic and morbidity profile. There were inequalities in hospitalization by socioeconomic status and gender. Patients with low contributory pensions and women had a lower risk of hospitalization. Diabetes mellitus, heart failure, and chronic kidney disease were associated with a higher risk of hospitalization. On the contrary, people with dementia showed the highest risk of mortality with no hospitalization. Patient-specific factors must be considered to develop equitable and effective measures in nursing homes to be prepared for future health threats.

## Introduction

In March 2020, the coronavirus disease 2019 (COVID-19) outbreak in China was declared a global pandemic (1). From that day, and according to the World Health Organization COVID-19 Dashboard (2), by January 2022, more than 315 million confirmed cases have been diagnosed worldwide. In Spain, almost 8 million cases have been declared and more than 90,000 people have died (3) in an unprecedented public health crisis.

One of the facts that the pandemic has brought to light is its greater impact on vulnerable groups. Inequalities have been observed in the risk of COVID-19 disease, with a higher risk of infection in groups with worse socioeconomic conditions. COVID-19 infection has shown a socioeconomic gradient, which has been linked to the type of job, the existence of lower health literacy or higher exposure rates, among others (4–7). This vulnerability has also been associated with the area of residence, due to household crowding and the existence of chronic stressors (8, 9), and both, individual and area vulnerability, mutually potentiate each other (10). These differences are not only limited to the risk of infection but also to the diagnosis of the disease and the medical attention received by these patients. Access to diagnostic tests (11) and to healthcare attention (12) seems to be worse for those people living in deprived areas, even in the universal healthcare systems. This may result in poorer care for the most vulnerable groups, amplifying existing inequalities.

The elderly population has been the most affected by the COVID-19 pandemic, especially in terms of mortality. Among the elderly, institutionalized people residing in nursing homes have been a particularly vulnerable group, showing high rates of infection and death in the 1 month of the pandemic and before the appearance of vaccines (13). The greatest impact of the COVID-19 pandemic on this group has been associated with both physical and psychological vulnerability, as well as with the living conditions related to the fact of residing in an institution (14, 15). In Spain, this fact has been particularly serious, as it is an aging country, with an aging index in 2020 of 125.75% (125 people aged over 64 years for every 100 aged under 16 years) (16). In addition, more than 300,000 elderly people live in nursing homes (17), where the effect of the pandemic was devastating: it is estimated that, only during the first wave, around 20,000 institutionalized people died, and the mortality rate for elderly people living in long-term care (LTC) facilities was 6% (18). These high mortality rates have been associated with high levels of community transmission and deficient nursing homes-related policy responses (19).

When analyzing COVID-19 mortality in nursing homes, factors, such as the patient's complex chronic conditions, the location, or the capacity of the center, have been analyzed (20, 21). Nonetheless, other aspects, such as the determinants of hospital admission or the existence of socioeconomic inequalities, are still unknown. Therefore, gaining a broad view of the factors involved in mortality and the healthcare received by these patients is an unavoidable task to prevent its recurrence. To this end, the objective of this study was to describe the profile of institutionalized patients with a confirmed COVID-19 infection in Aragón (Spain) and the socioeconomic and morbidity factors associated with hospitalization and death.

## Materials and Methods

### Design, Information Sources, and Study Population

Retrospective cohort study data were obtained from the Aragón-COVID-19 cohort. This is a health data collection of all individuals undergoing COVID-19 testing in the Spanish region of Aragón, an Autonomous Community in the northeastern Spain with a high aging rate 21.7% of people over 64 years of age (22).

The Aragón-COVID-19 cohort includes information gathered from administrative health data sources as well as electronic health records of the Aragón Health Service. The people included in the cohort were tested either when they presented symptoms compatible with COVID-19 or when they had close contact with a confirmed subject. All COVID-19 cases were confirmed by polymerase chain reaction (PCR) or COVID antigen testing. Individuals in the cohort were included from 9, March, 2020, the first epidemiological week with COVID-19 cases reported in Aragón, to 14, March, 2021, the end of the fourth wave in Aragón. On this date, 103,281 people were COVID-19 confirmed cases.

**Figure 1:**
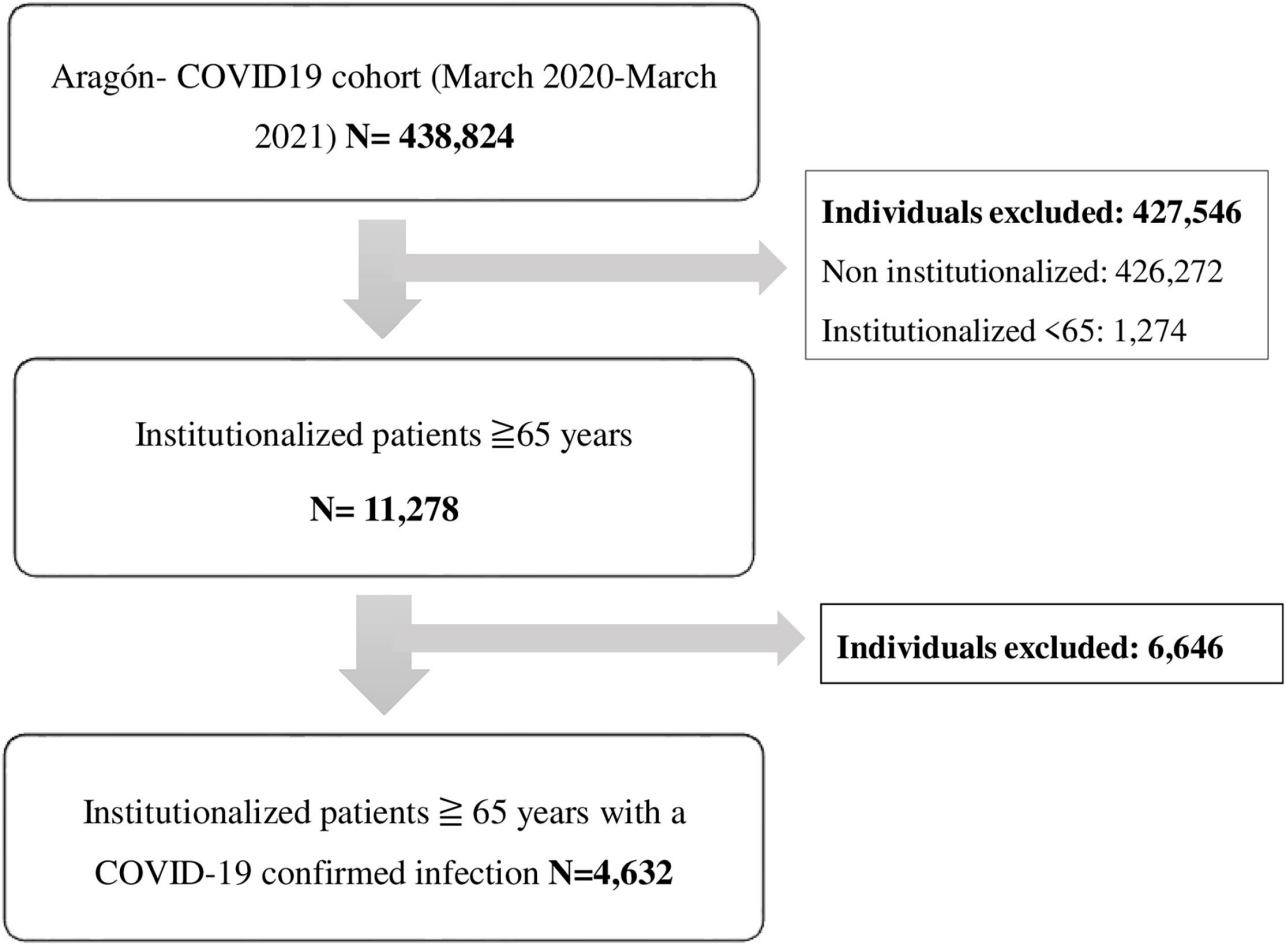
Study flowchart.

For this study, we selected subjects aged 65 years or older residing in a nursing home with a confirmed COVID-19 infection. This information was obtained from the Aragón health service user database (BDU) (Figure 1).

The research protocol of this study was approved by The Clinical Research Ethics Committee of Aragón (CEICA) (PI20/184).

### Variables of the Study

We considered the sociodemographic and clinical information of all the institutionalized individuals in the Aragón-COVID-19 cohort with a COVID-19 confirmed infection.

Regarding sociodemographic characteristics, we considered sex, age (65–79 years; ≥80 years of age), and socioeconomic level. The socioeconomic level was calculated on the basis of pharmacy copayment levels and social security benefits received, according to the type of user of the Aragón health service. From the combination of these two variables, 5 mutually exclusive categories were obtained for institutionalized patients as follows: individuals with a contributory pension <18,000€ per year; individuals with a contributory pension ≥ 18,000€ per year; individuals affiliated with the mutual insurance system for civil servants; individuals receiving free medicines (people with minimum integration income or who no longer receive the unemployment allowance); and other situations not previously considered.

Information related to the patient's clinical status was obtained from the morbidity-adjusted groups (GMA) (23). This source of information considers all medical diagnoses available in primary healthcare and hospitalization (hospital discharge records (CMBD) and emergency service). We considered GMA information from January 2020 in order to know the health status prior to the COVID-19 diagnosis of the individuals. The variables analyzed from GMA were weight complexity (obtained from the aggregation of the patient's different diagnoses); number of chronic morbidities; and existence of a medical diagnosis of diabetes mellitus, obesity, hypertension, stroke, ischemic heart disease, heart failure, chronic obstructive pulmonary disease (COPD), chronic kidney disease, depression, or dementia. These medical diagnoses were selected due to their high prevalence in this group of age.

The outcomes evaluated in patients with a COVID-19 confirmed infection were hospitalization and mortality by all causes. Only hospitalizations occurring within 14 days before and after COVID-19 diagnosis were considered in the study. In addition, since the cause of death was not available, we considered mortality from 3 days before diagnosis (as some patients died before the results of the test were obtained) to 90 days after. Both variables were obtained from the basic minimum dataset of hospital discharge (CMBDH) of Aragón.

### Analyses

First, we described the sociodemographic and clinical characteristics of all the individuals, over 64 years of age, living in a nursing home in Aragón with a confirmed diagnosis of COVID-19. In addition, a description of the sociodemographic and clinical profiles of the patients according to their hospitalization and mortality was conducted. To evaluate possible differences in the factors associated with mortality, this outcome was categorized into three different categories, namely, mortality at 7, 30, and 90 days after diagnosis. Categorical variables were described by percentages. Weight complexity and number of diagnoses had a non-normal distribution, so median and interquartile ranges were used to describe these variables. Statistical differences between categories were assessed using chi-square and Mann–Whitney *U*–tests.

To find out which sociodemographic and clinical characteristics were associated with the risk of hospitalization and death in institutionalized patients, univariate and multivariate logistic regression analyses were conducted. We performed explanatory logistic regression models. These models were adjusted by those available variables that were associated with hospitalization and death in the literature.

All analyses were performed using the R Statistical Software (the R Foundation for Statistical Computing, Vienna, Austria).

## Results

We identified 4,632 people aged 65 years or older residing in a nursing home with a COVID-19 confirmed infection in Aragón from March 2020 to March 2021. The description of the subjects included in the study according to their socioeconomic and clinical conditions, and their differences by sex, can be consulted online in Supplementary Table S1. They were mainly over 80 years of age, were pensioners with <18,000€ per year, and presented a high number of diseases. Hypertension (72.14%) and dementia (33.30%) were the most frequent diagnoses. Differences were observed between men and women for all the characteristics evaluated, with the only exception of the frequency of chronic kidney disease.

A total of 1,772 COVID-19 confirmed cases were hospitalized (38.3%) within 14 days of COVID-19 diagnosis. Results are summarized in Table 1. Hospitalization was slightly more frequent in men than in women (*p* <0.001) and in people with a contributory pension of 18,000€ per year or more (*p* <0.001). Regarding clinical diagnoses, people residing in a nursing home with a diagnosis of DM, ischemic heart disease, heart failure, COPD, or chronic kidney disease showed a higher frequency of hospitalization. No statistical differences were observed by age groups and a diagnosis of obesity, stroke, hypertension, depression, or dementia. In 145 individuals (109 women and 36 men), no previous morbidity was recorded.

**Table 1:** Hospitalization in COVID-19 confirmed institutionalized patients over 64 years of age.

	**Global (*N* = 4,632)**	**No hospitalization (*N* = 2,860)**	**Hospitalization (*N* = 1,772)**	***p***
Sex				<0.001*
Male	1,621 (35.00%)	865 (30.24%)	756 (42.66%)	
Female	3,011 (65.00%)	1,995 (69.76%)	1,016 (57.34%)	
Age				0.058
65–79	760 (16.41%)	493 (17.24%)	267 (15.07%)	
≥80	3,872 (83.59%)	2,367 (82.76%)	1,505 (84.93%)	
Socioeconomic level				<0.001*
Mutualist	160 (3.45%)	121 (4.23%)	39 (2.20%)	
Pensioner <18,000€/year	3,639 (78.56%)	2,260 (79.02%)	1,379 (77.82%)	
Pensioner ≥ 18,000€/year	611 (13.19%)	337 (11.78%)	274 (15.46%)	
Free medicines	184 (3.97%)	114 (3.99%)	70 (3.95%)	
Other	38 (0.82%)	28 (0.98%)	10 (0.56%)	
Number of diseases (a)	6.00 (4.00; 8.00)	6.00 (4.00; 8.00)	6.00 (5.00; 8.00)	<0.001*
Complexity (a)	3.00 (2.00; 4.00)	3.00 (2.00; 4.00)	3.00 (2.00; 4.00)	0.003*
Diagnosis	
Diabetes mellitus	1,166 (25.99%)	668 (24.31%)	498 (28.64%)	0.001*
Obesity	440 (9.50%)	343 (12.48%)	229 (13.17%)	0.531
Hypertension	3,237 (72.14%)	1,966 (71.54%)	1,271 (73.09%)	0.275
Stroke	654 (14.58%)	382 (13.90%)	272 (15.64%)	0.117
Ischemic heart disease	460 (10.25%)	254 (9.24%)	206 (11.85%)	0.006*
Heart failure	578 (12.88%)	321 (11.68%)	257 (14.78%)	0.003*
COPD	453 (10.10%)	243 (8.84%)	210 (12.08%)	0.001*
Chronic kidney disease	1,258 (28.04%)	716 (26.06%)	542 (31.17%)	<0.001*
Depression	1,269 (28.28%)	771 (28.06%)	498 (28.64%)	0.699
Dementia	1,494 (33.30%)	928 (33.77%)	566 (32.55%)	0.416

*Sociodemographic and clinical characteristics.*

*N, number; p, statistical significance; a, results expressed as median and interquartile range; COPD, chronic obstructive pulmonary disease.*

*Statistically significant results.

We evaluated mortality within 7, 30, and 90 days after COVID-19 diagnosis. In Table 2, results related to the socioeconomic and clinical profiles of both dead and alive patients for each cutoff point are available.

**Table 2:** Mortality in COVID-19 confirmed institutionalized patients over 64 years of age.

		**Mortality at 7 days**			**Mortality at 30 days**			**Mortality at 90 days**		
		**Alive (*N* = 4,292)**	**Death (*N* = 340)**	***p***	**Alive (*N* = 3,504)**	**Death (*N* = 1,128)**	***P***	**Alive (*N* = 3,174)**	**Death (*N* = 1,458)**	***p***
Sex:	Male	1,478 (34.44%)	143 (42.06%)	0.005*	1,162 (33.16%)	459 (40.69%)	<0.001*	1,034 (32.58%)	587 (40.26%)	<0.001*
	Female	2,814 (65.56%)	197 (57.94%)		2,342 (66.84%)	669 (59.31%)		2,140 (67.42%)	871 (59.74%)	
Age:	65–79	726 (16.92%)	34 (10.00%)	0.001*	657 (18.75%)	103 (9.13%)	<0.001*	620 (19.53%)	140 (9.60%)	<0.001*
	≥80	3,566 (83.08%)	306 (90.00%)		2,847 (81.25%)	1,025 (90.87%)		2,554 (80.47%)	1,318 (90.40%)	
SE level:	Mutualist	153 (3.56%)	7 (2.06%)	0.328	138 (3.94%)	22 (1.95%)	0.003*	130 (4.11%)	30 (2.05%)	0.001*
	Pensioner <18,000€/year	3,359 (78.26%)	280 (82.35%)		2,748 (78.42%)	891 (78.99%)		2,468 (77.76%)	1,171 (80.32%)	
	Pensioner ≥18,000€/year	573 (13.35%)	38 (11.18%)		448 (12.79%)	163 (14.45%)		415 (13.07%)	196 (13.44%)	
	Free medicines	170 (3.96%)	14 (4.12%)		136 (3.88%)	48 (4.26%)		128 (4.04%)	56 (3.82%)	
	Other	37 (0.86%)	1 (0.29%)		34 (0.97%)	4 (0.35%)		33 (1.04%)	5 (0.34%)	
Number of diseases (a)		6.00 (4.00;8.00)	6.00 (5.00;8.00)	0.012*	6.00 (4.00;8.00)	6.00 (5.00;8.00)	0.003*	6.00 (4.00;8.00)	6.00 (5.00;8.00)	0.004*
Complexity (a)		3.00 (2.00;4.00)	3.00 (2.00;4.00)	0.015*	3.00 (2.00;4.00)	3.00 (2.00;4.00)	<0.001*	3.00 (2.00;4.00)	3.00 (2.00;4.00)	0.003*
Diagnosis:	Diabetes mellitus	1,068 (25.72%)	98 (29.25%)	0.176	850 (25.20%)	316 (28.37%)	0.040*	771 (25.30%)	395 (27.45%)	0.134
	Obesity	535 (12.89%)	37 (11.04%)	0.375	448 (13.28%)	124 (11.13%)	0.070	410 (13.45%)	162 (11.26%)	0.045*
	Hypertension	3,001 (72.28%)	236 (70.45%)	0.512	2,453 (72.72%)	784 (70.38%)	0.140	2,216 (72.70%)	1,021 (70.95%)	0.236
	Stroke	599 (14.43%)	55 (16.42%)	0.361	475 (14.08%)	179 (16.07%)	0.114	427 (14.01%)	227 (15.77%)	0.129
	Ischemic heart disease	419 (10.09%)	41 (12.24%)	0.249	315 (9.34%)	145 (13.02%)	0.001*	293 (9.61%)	167 (11.61%)	0.045*
	Heart failure	514 (12.38%)	64 (19.10%)	0.001*	399 (11.83%)	179 (16.07%)	<0.001*	359 (11.78%)	219 (15.22%)	0.002*
	COPD	412 (9.92%)	41 (12.24%)	0.208	321 (9.52%)	132 (11.85%)	0.029*	281 (9.22%)	172 (11.95%)	0.005*
	Chronic kidney disease	1,147 (27.63%)	111 (33.13%)	0.036*	894 (26.50%)	364 (32.68%)	<0.001*	797 (26.15%)	461 (32.04%)	<0.001*
	Depression	1,167 (28.11%)	102 (30.45%)	0.394	949 (28.14%)	320 (28.73%)	0.733	867 (28.44%)	402 (27.94%)	0.751
	Dementia	1,372 (33.04%)	122 (36.42%)	0.230	1,086 (32.20%)	408 (36.62%)	0.007*	968 (31.76%)	526 (36.55%)	0.002*

*Sociodemographic and clinical characteristics.*

*N, number; SE socioeconomic; p, statistical significance; a, results expressed as median and interquartile range; COPD, chronic obstructive pulmonary disease.*

*Statistically significant results.

A total of 1,458 people aged 65 years or older residing in a nursing home with a confirmed COVID-19 infection in Aragón died within 90 days of COVID-19 diagnosis from all causes (31.5%). Mortality in men and in people aged 80 years or older was higher for the three time intervals considered. Differences in socioeconomic status were observed at 30 and 90 days.

Regarding morbidity, mortality increased in people with a high number of diseases and with high complexity for all the time intervals evaluated. Mortality was higher for the three moments evaluated for heart failure and chronic kidney disease. A higher risk of death at 30 and 90 days of COVID-19 diagnosis was also observed in people with ischemic heart disease, COPD, and dementia. In contrast, people with obesity showed a lower mortality at 90 days (*p* = 0.045).

We analyzed those COVID-19 confirmed institutionalized patients who died within 90 days after diagnosis and their probability of having been hospitalized by COVID-19 (Table 3). Of the 1,458 patients who died, 523 (35.8%) patients were not hospitalized by COVID-19. Differences in hospitalization were observed according to sex. Those women who died showed a lower prevalence of hospitalization than men (*p* <0.001). People who died with a high number of chronic diseases, diabetes mellitus and heart failure were more frequently hospitalized. However, people who died with dementia showed a lower probability of hospitalization (*p* <0.001).

**Table 3:** Hospitalization in COVID-19 confirmed institutionalized patients over 64 years of age who died.

	**Mortality at 90 days (*N* = 1,458)**	**No hospitalization (*N* = 523)**	**Hospitalization (*N* = 935)**	***p***
Sex				0.001*
Male	587 (40.26%)	181 (34.61%)	406 (43.42%)	
Female	871 (59.74%)	342 (65.39%)	529 (56.58%)	
Age				0.072
65–79	140 (9.60%)	40 (7.65%)	100 (10.70%)	
≥80	1,318 (90.40%)	483 (92.35%)	835 (89.30%)	
Socioeconomic level				0.247
Mutualist	30 (2.06%)	11 (2.10%)	19 (2.03%)	
Pensioner <18,000€/year	1,171 (80.32%)	435 (83.17%)	736 (78.72%)	
Pensioner ≥ 18,000€/year	196 (13.44%)	57 (10.90%)	139 (14.87%)	
Free medicines	56 (3.84%)	18 (3.44%)	38 (4.06%)	
Other	5 (0.34%)	2 (0.38%)	3 (0.32%)	
Number of diseases (a)	6.00 [5.00;8.00]	6.00 [4.00;8.00]	6.00 [5.00;8.00]	0.022 *
Complexity (a)	3.00 [2.00;4.00]	3.00 [2.00;4.00]	3.00 [2.00;4.00]	0.774
Diagnosis				
Diabetes mellitus	395 (27.45%)	116 (22.66%)	279 (30.10%)	0.003*
Obesity	162 (11.26%)	46 (8.98%)	116 (12.51%)	0.052
Hypertension	1,021 (70.95%)	357 (69.73%)	664 (71.63%)	0.484
Stroke	227 (15.77%)	76 (14.84%)	151 (16.29%)	0.519
Ischemic heart disease	167 (11.61%)	50 (9.77%)	117 (12.62%)	0.125
Heart failure	219 (15.22%)	60 (11.72%)	159 (17.15%)	0.008*
COPD	172 (11.95%)	57 (11.13%)	115 (12.41%)	0.530
Chronic kidney disease	461 (32.04%)	157 (30.66%)	304 (32.79%)	0.441
Depression	402 (27.94%)	142 (27.73%)	260 (28.05%)	0.948
Dementia	526 (36.55%)	224 (43.75%)	302 (32.58%)	<0.001*

*Sociodemographic and clinical characteristics.*

*N, number; p, statistical significance; a, results expressed as median and interquartile range; COPD, chronic obstructive pulmonary disease.*

*Statistically significant results.

We conducted multivariate models to analyze those factors associated with the risk of hospitalization by COVID-19 and death at 7, 30, and 90 days in our population (Table 4). Women showed a lower risk of hospitalization and death than men. The risk of hospitalization and death was also higher in people aged 80 years or older than in those aged 65–79 years. Regarding socioeconomic status, people with a contributory pension of €18,000 or more showed a higher risk of hospitalization than those with low contributory pensions [odds ratio (OR): 1.24; 95% CI 1.04–1.48]. No differences were observed according to death. Finally, the number of chronic diagnoses was associated with a higher risk of hospitalization and death at 7 and 90 days. High complexity was only associated with a higher risk of death at 30 days (*p* = 0.004).

**Table 4:** Sociodemographic and clinical factors associated with hospitalization and mortality in COVID-19 confirmed institutionalized patients over 64 years of age.

		**Hospitalization**		**Mortality at 7 days**		**Mortality at 30 days**		**Mortality at 90 days**	
		**OR (95%CI)**	***P***	**OR (95%CI)**	***P***	**OR (95%CI)**	***p***	**OR (95%CI)**	***p***
Sex:	Male	*Reference*		*Reference*		*Reference*		*Reference*	
	Female	0.57 (0.50–0.64)	<0.001*	0.69 (0.54–0.87)	0.002*	0.68 (0.59–0.79)	<0.001*	0.66 (0.57–0.75)	<0.001*
Age:	65–79	*Reference*		Reference		Reference		Reference	
	≥ 80	1.27 (1.07–1.51)	0.006*	*1.90 (1.33–2.81)*	0.001*	*2.43 (1.95–3.06)*	<0.001*	*2.39 (1.96–2.93)*	<0.001*
Socioeconomic level:	Pensioner <18,000€	Reference		Reference		Reference		Reference	
	Mutualist	0.91 (0.50–1.62)	0.754	1.73 (0.65–3.82)	0.219	1.24 (0.64–2.29)	0.498	1.23 (0.67–2.19)	0.495
	Pensioner ≥ 18,000€	1.24 (1.04–1.48)	0.019*	0.77 (0.53–1.08)	0.143	1.09 (0.89–1.33)	0.376	0.97 (0.80–1.17)	0.729
	Free medicines	1.14 (0.83–1.55)	0.414	1.18 (0.64–2.00)	0.574	1.33 (0.93–1.87)	0.106	1.13 (0.81–1.56)	0.475
	Other	0.72 (0.31–1.49)	0.394	0.48 (0.03–2.25)	0.471	0.54 (0.16–1.38)	0.249	0.46 (0.16–1.10)	0.113
Number of diseases		1.05 (1.03–1.08)	<0.001*	1.05 (1.01–1.09)	0.010*	1.02 (1.00–1.05)	0.051	1.03 (1.00–1.05)	0.027*
Complexity		1.01 (0.96–1.07)	0.682	1.07 (0.96–1.18)	0.231	1.10 (1.03–1.17)	0.004*	1.05 (0.99–1.11)	0.116

*Adjusted results.*

*OR, Odds ratios; 95%CI, 95% Confidence interval; p, statistical significance.*

*Statistically significant results.

*Odds ratios adjusted by sex, age, socioeconomic level, number of diseases and complexity*.

**Figure 2:**
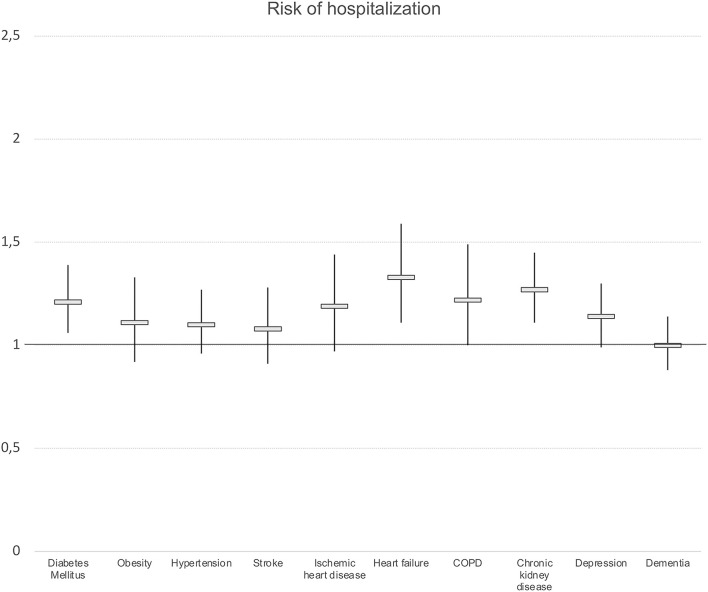
Presence of chronic morbidity and risk of hospitalization. Logistic regression models. Odds ratios and 95%Confidence intervals. Results adjusted by sex, age and socioeconomic level. COPD, chronic obstructive pulmonary disease.

**Figure 3:**
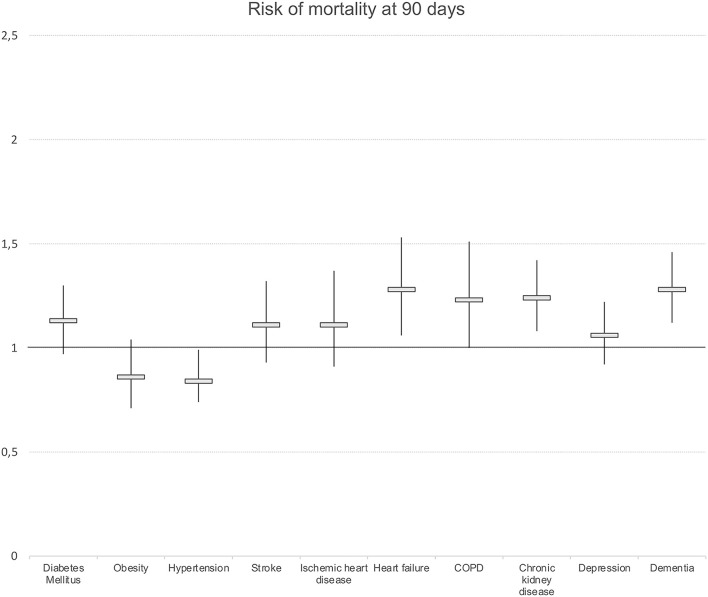
Presence of chronic morbidity and risk of mortality at 90 days. Logistic regression models. Odds ratios and 95%Confidence intervals. Results adjusted by sex, age and socioeconomic level. COPD, chronic obstructive pulmonary disease.

We observed differences in the risk of hospitalization and mortality risk according to chronic morbidity. The existence of DM, heart failure, and chronic kidney insufficiency was associated with a higher risk of hospitalization (Figure 2). Regarding mortality, heart failure was associated with a higher risk of mortality for all the cutoff points considered (OR: 1.62; 95% CI 1.20–2.15 at 7 days). Other diagnoses associated with a higher risk of mortality at 90 days were chronic kidney disease (OR: 1.24; 95% CI 1.08–1.42) and dementia (OR: 1.28; 95% CI 1.12–1.46) (Figure 3).

**Figure 4:**
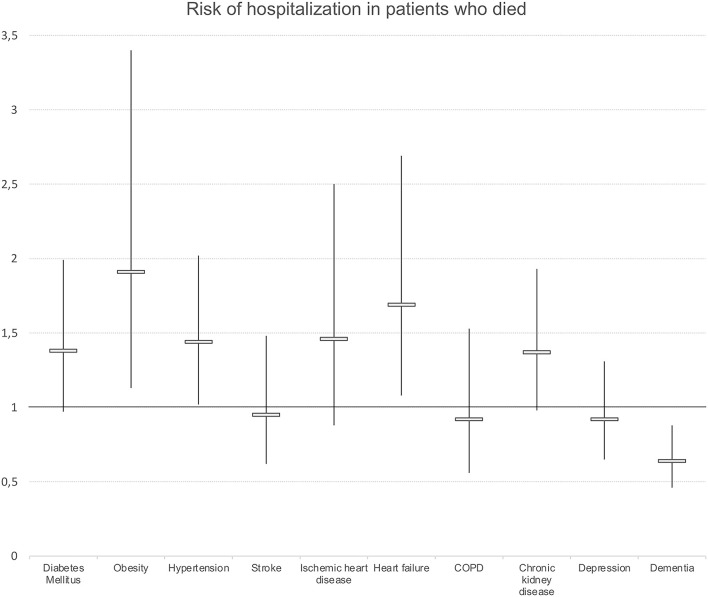
Presence of chronic morbidity and risk of hospitalization in those who died at 90 days. regression models. Odds ratios and 95%Confidence intervals. Results adjusted by sex, age and socioeconomic level. COPD, chronic obstructive pulmonary disease.

When we analyzed the risk of hospitalization in those who died of any cause at 90 days, multivariate analyses showed that the risk of hospitalization was lower in women than in men (OR: 0.67; 95% CI 0.53–0.84). An increasing number of diseases were associated with a high risk of hospitalization (OR: 1.07; 95% CI 1.03–1.11). No differences were observed by age, complexity, or socioeconomic position. We also observed differences in hospitalization in patients who died according to chronic morbidity. The diagnoses of DM, obesity, and heart failure were associated with a higher risk of hospitalization. On the contrary, a diagnosis of dementia was associated with a lower risk of hospitalization (OR: 0.64; 95% CI 0.51–0.80) (Figure 4).

## Discussion

In Aragón, 38.3% of COVID-19 confirmed patients over 64 years of age residing in a nursing home were hospitalized. The risk of hospitalization varied according to sociodemographic and morbidity profiles. Therefore, the risk of hospitalization was higher in men and in older people. Those with a contributory pension equal to or > 18,000€ per year showed a slightly higher risk of hospitalization than those with lower pensions. People with a diagnosis of DM, heart failure, or chronic kidney disease also showed a higher risk of hospitalization.

Of all COVID-19 confirmed patients residing in a nursing home, 31.5% died at 90 days of COVID-19 diagnosis. Mortality was higher in men and in older patients. Heart failure was the diagnosis showing a stronger association with the risk of death. Finally, 35.8% of the residents with a COVID-19 confirmed diagnosis who died had not been hospitalized. Hospitalization in those patients who died was positively associated with being men and a diagnosis of DM, obesity, or heart failure. On the contrary, patients with dementia showed a higher risk of mortality without hospitalization.

COVID-19 has had a devastating impact on old people residing in nursing homes. In Aragón, almost 40% of the patients required hospitalization and one of three patients died. Some personal factors have been associated with the vulnerability of these subjects as follows: the existence of frailty patients (24), low Barthel index, or the high prevalence of comorbidities (25, 26). In addition, organizational factors have been involved in this equation. A large number of beds in many LTC facilities, the very low staffing ratios, shortage of qualified professionals, or the deficient coordination between social and health services (27) are some of the factors that can explain the high impact of the COVID-19 pandemic in Spanish nursing homes.

There is a relationship between the sociodemographic characteristics of the patients with a COVID-19 confirmed diagnosis living in a nursing home and their risk of hospitalization and death. Men showed a higher risk of hospitalization than women, as well as a higher risk of death, after adjusting by age, socioeconomic position, number of chronic diseases, and complexity. This fact has already been described widely in the literature (28, 29) and has been related to biological, psychosocial, and behavioral factors (30). However, it is striking that among those patients who died, women also had a lower risk of being hospitalized. Another study conducted in Spain on the general population (31) found that women presented different symptoms at disease onset, clinical outcomes, and treatment patterns, with differences in hospitalization and intensive care unit admission. Further research is required to explore the factors that could have conditioned this gender bias.

We also found differences in hospitalization according to socioeconomic level but not for mortality risk. Those old patients living in a nursing home with a contributory pension equal to or higher than 18,000€ per year had a higher risk of hospitalization than those with lower pensions, after taking into account age, sex, and morbidities. When we selected those people who died at 90 days of diagnosis, there were no differences in hospitalization by socioeconomic status, but differences existed when considering mortality at 7 days (OR: 4.5; 95% CI 1.9–12.6). Nonetheless, when analyzing the profile of those patients who survived, people with a contributory pension of 18,000€ per year or higher had a high risk of hospitalization (*p* = 0.046). It has been described the association between low socioeconomic status and a higher risk of hospitalization and death by COVID-19 in the general population (12, 32) but, to the best of our knowledge, this is the first study to assess the influence of individual socioeconomic status on the risk of hospitalization and death from COVID-19 in institutionalized patients. A poor individual socioeconomic level may reflect deficient conditions of the nursing homes, which could result in poorer care for these patients, but also the existence of few social and support networks.

The suffering of some chronic diseases was associated with hospitalization and death. In this sense, patients with heart failure had the highest risk of hospitalization and death, after controlling by sex, age, and socioeconomic position. Our results are consistent with other studies, in which patients with underlying cardiovascular disease have an increased risk of mechanical ventilation and death by COVID-19 (33, 34). In the case of COVID-19 infection in patients with this illness, it seems to be associated with a significant risk of developing acute decompensation (35). Patients with heart failure have also shown an increased risk of COVID-19 infection due to reduced immunity, frailty, and low hemodynamic ability to cope with severe infections (36). In contrast, people with dementia had the highest risk of mortality with no hospitalization. Lockdown and quarantine have had a high impact on patients with dementia living in nursing homes. Changes in their routines and physical inactivity lead to a worsening of their functional and cognitive status (37) and an increased stress in an already vulnerable population (38), resulting in a high risk of mortality by COVID-19 (39, 40). Some of the reasons proposed to explain this fact were the advanced age of these patients and the existence of comorbidities. Nonetheless, in our study, a high risk of mortality at 90 days in people with dementia was observed even after adjusting by the presence of other comorbidities. Other authors have pointed out the presence of atypical symptoms of infection (41), namely, the onset of hypoactive delirium and worsening functional status (42), as the cause of an increased mortality in this group. This atypical presentation could explain the lower risk of hospitalization observed in patients with dementia and COVID-19 who died.

This study has several strengths. We analyzed all the individuals residing in a nursing home with a confirmed COVID-19 infection from the population of Aragón, including data from administrative health data sources and electronic health records. Clinical diagnoses were obtained from GMA. This source of information combines diagnoses from primary healthcare and from hospital admissions, which makes this a high-sensitivity classification. Finally, we used a combination of two different socioeconomic indicators (pharmacy copayment levels and the type of user of the Aragón Health Service) to categorize the socioeconomic level of the individuals. The combination of these two variables has already been used in other analyses of health inequities at a population level (10, 43) and provides a good knowledge of the individual socioeconomic position.

Nevertheless, this study has some limitations. There are limitations inherent to observational studies, such as quality of data and cases with incomplete data. Second, neither the cause of death nor the cause of hospitalization was available. The cause of death was not identified because the information from the Aragón-COVID-19 cohort could not be matched with the information available in the mortality registry. To address this issue, only deaths occurring up to 90 days after COVID-19 diagnosis were considered. A total of 481 institutionalized patients over 64 years of age died after 90 days of COVID-19 diagnosis, with a median of 207 days. In addition, we only considered hospital admissions within 14 days, both before and after COVID-19, as the hospital discharge records (CMBD), where hospital cause is codified, were not available in the Aragón-COVID-19 cohort. In this case, 218 patients were hospitalized but did not fulfill our criteria, with a median of −21 days. Instead of the possible bias, we considered that the established criteria allow us to define plausible ranges for identifying both death and hospitalization due to COVID-19. Finally, some of the patients who were not hospitalized could have been treated in one of the “COVID centers” set up in Aragón in the first waves of the pandemic. This information was not available for its consideration.

## Conclusion

Many challenges have been faced by nursing homes in this COVID-19 pandemic. The characteristics of its residents and the delay of the measures taken have had a devastating effect in terms of morbidity and mortality. In this study, we found gender and socioeconomic inequalities in the risk of hospitalization of these patients, as well as an increased risk of hospitalization and death for some diagnostic groups.

The LTC facilities must be prepared for future health threats, and this requires an appropriate implementation of geriatric interventions (44) and taking into account patient-specific factors, in order to develop equitable and effective measures. As we have observed in our analyses, patients with underlying cardiac pathologies may require special attention, given their potential severity. In contrast, people with dementia showed the highest risk of mortality with no hospitalization. In this group of patients, a strict medical support and control (39) or the implementation of applications to promote interaction with family members (38) is necessary. Finally, the professionals involved should be aware of the existence of gender and socioeconomic biases when assessing and caring for patients, in order to avoid adopting measures that contribute to increase the existing inequalities.

## Data Availability Statement

Aragon-COVID19 data is available under request to IACS. Requests to access these datasets should be directed to https://www.iacs.es.

## Ethics Statement

The studies involving human participants were reviewed and approved by Clinical Research Ethics Committee of Aragón (CEICA). Written informed consent for participation was not required for this study in accordance with the national legislation and the institutional requirements.

## Author Contributions

All authors listed have made a substantial, direct, and intellectual contribution to the work and approved it for publication. All authors contributed to the article and approved the submitted version.

## Funding

This research was funded by the Grupo de Investigacion en Servicios Sanitarios de Aragon (GRISSA) [B09-20R] of the IIS Aragon, and funded by the regional Government of Aragon, Spain (Decreto-ley 3/2020 del Gobierno de Aragón; Orden CUS/1166/2020).

## Conflict of Interest

The authors declare that the research was conducted in the absence of any commercial or financial relationships that could be construed as a potential conflict of interest.

## Publisher's Note

All claims expressed in this article are solely those of the authors and do not necessarily represent those of their affiliated organizations, or those of the publisher, the editors and the reviewers. Any product that may be evaluated in this article, or claim that may be made by its manufacturer, is not guaranteed or endorsed by the publisher.
