# Supplementary material for: Understanding the COVID-19 Pandemic in Nursing Homes (Aragón, Spain): Sociodemographic and Clinical Factors Associated With Hospitalization and Mortality
Source: Front Public Health. 2022 Jul 7;10:928174. doi: 10.3389/fpubh.2022.928174 (PMC9301241; doi:10.3389/fpubh.2022.928174)
Supplement: Supplementary file 1 [file Table_1.DOCX]

Supplementary Material

# Supplementary Table

**Supplementary table S1.** Sociodemographic and clinical characteristics of COVID-19 confirmed institutionalized patients over 64 years of age.

|  | **Global**  **(N= 4,632)** | **Men**  **(N= 1,621)** | **Women**  **(N= 3,011)** | **P*** |
| --- | --- | --- | --- | --- |
| **Age** |  |  |  | <0.001* |
| **65-79** | 760 (16.41%) | 372 (22.95%) | 388 (12.89%) |  |
| **>=80** | 3872 (83.59%) | 1249 (77.05%) | 2623 (87.11%) |  |
| **Socioeconomic level** |  |  |  | <0.001* |
| **Mutualist** | 160 (3.45%) | 45 (2.78%) | 115 (3.82%) |  |
| **Pensioner <€18,000€/year** | 3639 (78.56%) | 1228 (75.76%) | 2411 (80.07%) |  |
| **Pensioner ≥ €18,000€/year** | 611 (13.19%) | 307 (18.94%) | 304 (10.10%) |  |
| **Free medicines** | 184 (3.97%) | 34 (2.10%) | 150 (4.98%) |  |
| **Other** | 38 (0.82%) | 7 (0.43%) | 31 (1.03%) |  |
| **Number of diseases (a)** | 6.00 [4.00;8.00] | 6.00 [5.00;8.00] | 6.00 [4.00;8.00] | <0.001* |
| **Complexity (a)** | 3.00 [2.00;4.00] | 3.00 [2.00;4.00] | 3.00 [2.00;4.00] | <0.001* |
| **Diagnosis** |  |  |  |  |
| **Diabetes Mellitus** | 1166 (25.99%) | 477 (30.09%) | 689 (23.74%) | <0.001* |
| **Obesity** | 572 (12.75%) | 175 (11.04%) | 397 (13.68%) | 0.013* |
| **Hypertension** | 3237 (72.14%) | 1060 (66.88%) | 2177 (75.02%) | <0.001* |
| **Stroke** | 654 (14.58%) | 287 (18.11%) | 367 (12.65%) | <0.001* |
| **Ischemic heart disease** | 460 (10.25%) | 229 (14.45%) | 231 (7.96%) | <0.001* |
| **Heart failure** | 578 (12.88%) | 172 (10.85%) | 406 (13.99%) | 0.003* |
| **COPD** | 453 (10.10%) | 287 (18.11%) | 166 (5.72%) | <0.001* |
| **Chronic kidney disease** | 1258 (28.04%) | 432 (27.26%) | 826 (28.46%) | 0.409 |
| **Depression** | 1269 (28.28%) | 290 (18.30%) | 979 (33.74%) | <0.001* |
| **Dementia** | 1494 (33.30%) | 389 (24.54%) | 1105 (38.08%) | <0.001* |

N: number; p: statistical significance; a: results expressed as median and interquartile range; COPD: chronic obstructive pulmonary disease. * statistically significant results.
